# Supplementary material for: The Construction and Exploration of a Comprehensive MicroRNA Centered Regulatory Network in Foxtail Millet (Setaria italica L.)
Source: Front Plant Sci. 2022 May 6;13:848474. doi: 10.3389/fpls.2022.848474 (PMC9121102; doi:10.3389/fpls.2022.848474)
Supplement: Supplementary file 3 [file Data_Sheet_3.zip › Sit-miRNovel2_Seita.9G077900.1_177_TPlot.pdf]

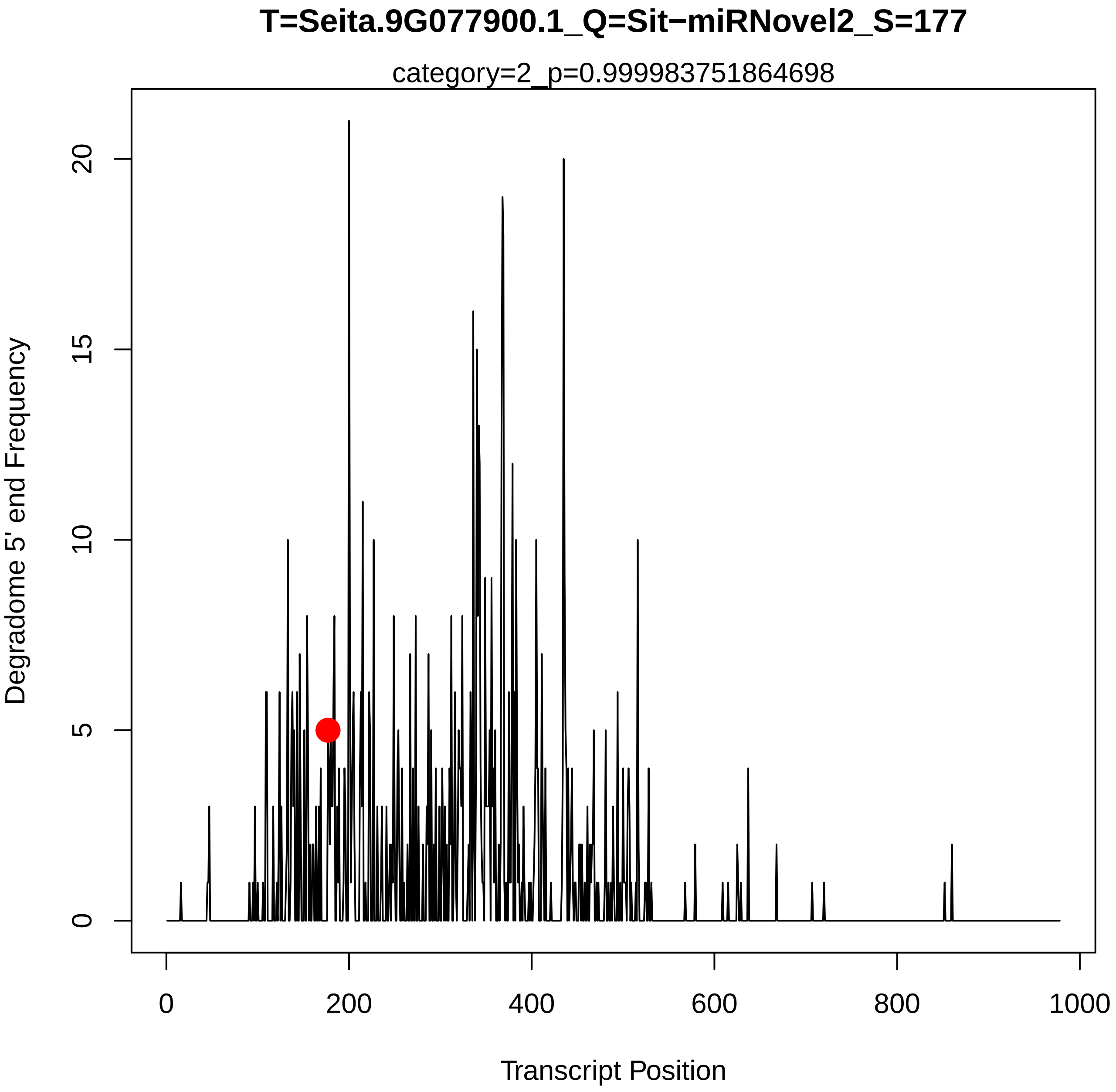

T=Seita.9G077900.1_Q=Sit−miRNovel2_S=177
category=2_p=0.999983751864698
Degradome 5' end Frequency
0
5
10
15
20
0
200
400
600
800
1000
Transcript Position
